# An observational analysis of frailty in combination with loneliness or social isolation and their association with socioeconomic deprivation, hospitalisation and mortality among UK Biobank participants

**DOI:** 10.1038/s41598-024-57366-7

**Published:** 2024-03-27

**Authors:** Marina Politis, Lynsay Crawford, Bhautesh D. Jani, Barbara I. Nicholl, Jim Lewsey, David A. McAllister, Frances S. Mair, Peter Hanlon

**Affiliations:** School of Health and Wellbeing, University of Glasgow, Clarice Pears Building, Byres Road, Glasgow, UK

**Keywords:** Frailty, Social isolation, Loneliness, Mortality, Hospitalisation, Epidemiology, Risk factors

## Abstract

Frailty, social isolation, and loneliness have individually been associated with adverse health outcomes. This study examines how frailty in combination with loneliness or social isolation is associated with socioeconomic deprivation and with all-cause mortality and hospitalisation rate in a middle-aged and older population. Baseline data from 461,047 UK Biobank participants (aged 37–73) were used to assess frailty (frailty phenotype), social isolation, and loneliness. Weibull models assessed the association between frailty in combination with loneliness or social isolation and all-cause mortality adjusted for age/sex/smoking/alcohol/socioeconomic-status and number of long-term conditions. Negative binomial regression models assessed hospitalisation rate. Frailty prevalence was 3.38%, loneliness 4.75% and social isolation 9.04%. Frailty was present across all ages and increased with age. Loneliness and social isolation were more common in younger participants compared to older. Co-occurrence of frailty and loneliness or social isolation was most common in participants with high socioeconomic deprivation. Frailty was associated with increased mortality and hospitalisation regardless of social isolation/loneliness. Hazard ratios for mortality were 2.47 (2.27–2.69) with social isolation and 2.17 (2.05–2.29) without social isolation, 2.14 (1.92–2.38) with loneliness and 2.16 (2.05–2.27) without loneliness. Loneliness and social isolation were associated with mortality and hospitalisation in robust participants, but this was attenuated in the context of frailty. Frailty and loneliness/social isolation affect individuals across a wide age spectrum and disproportionately co-occur in areas of high deprivation. All were associated with adverse outcomes, but the association between loneliness and social isolation and adverse outcomes was attenuated in the context of frailty. Future interventions should target people living with frailty or loneliness/social isolation, regardless of age.

## Introduction

Frailty, social isolation and loneliness are each rising in prevalence and are associated with a range of adverse health outcomes. Frailty describes a reduction in physiological reserve and increased vulnerability to decompensation due to poor resolution of homeostasis following stressors^1–3^, increasing the risk of adverse outcomes. These include falls, cardiovascular events, hospitalisation, and mortality^1–3^. There are multiple models of frailty, but two common measures are the frailty phenotype^4^ and frailty index^5^. While these measures were originally developed to assess frailty in older populations (generally > 65 years) several studies have shown that frailty also predicts adverse health outcomes such as mortality and hospitalisation when applied to younger populations^6^. The prevalence of frailty rises with age, affecting around 10% of people over 65, and over a third of those over 80^7^. Frailty is rarer, but does occur, in people in people younger than 65, particularly in the context of high socioeconomic deprivation^2,8^. Despite this growing literature, the implications of applying the frailty concept to younger populations have not been widely explored.

Both loneliness and social isolation describe aspects of social vulnerability, the accumulation of numerous, varied social problems with a bidirectional relationship on adverse health outcomes^9,10^. Loneliness refers to the subjective experience of feeling alone, i.e., perceived deficits in social connection^11^, whilst social isolation describes an objective lack of social connections, a condition of not having ties with others^12^. Like frailty, social isolation and loneliness are associated with socioeconomic deprivation and with adverse health outcomes^13^, including (but not limited to) cardiovascular events and mental health conditions^14,15^.

While loneliness and social isolation have been associated with adverse health and older individuals with high levels of loneliness are at increased risk of frailty^16–18^, the association between the combination of frailty and social isolation or loneliness with adverse outcomes is less clear^10,11,16^. Furthermore, the overlap between frailty and loneliness or social isolation, and their joint associations with socioeconomic deprivation, have not been explored among relatively younger people.

This study examines if frailty in combination with loneliness or social isolation is associated with adverse health outcomes (all-cause mortality and number of hospitalisations), using data from UK Biobank.

## Results

**Figure 1:**
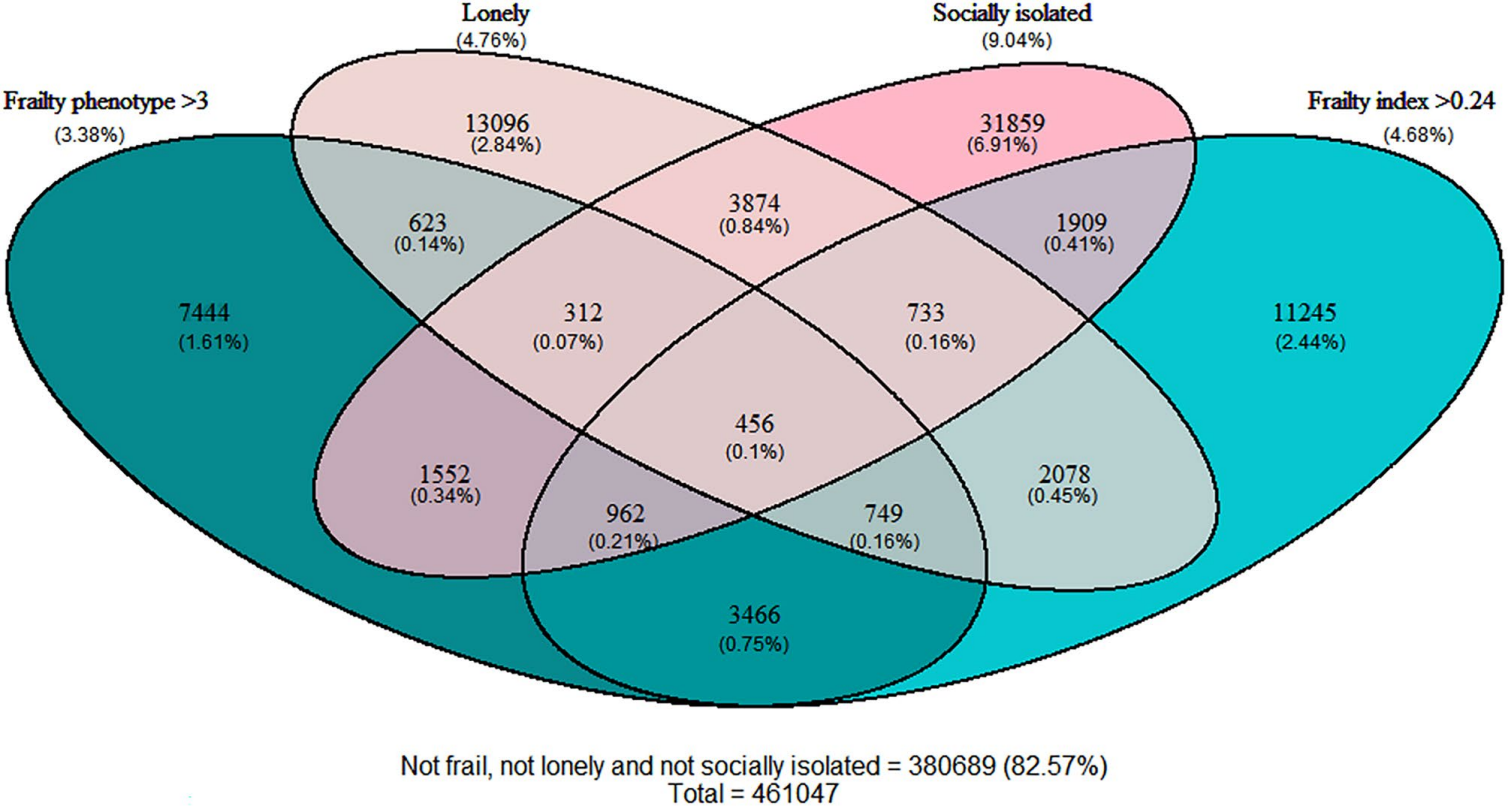
Overlap between frailty phenotype, frailty index, social isolation and loneliness. This diagram displays the number of participants with combinations of each frailty definition, loneliness or social isolation.

Of the sample of 502,456 participants in UK Biobank, 461,047 had complete data for frailty, loneliness, and social isolation (mean age 56.5, 251,604 (54.6%) female). Both loneliness and social isolation were more common among people with frailty (Table 1). Overlap between participants identified by each measure is shown in Fig. 1.

**Table 1:** Prevalence of frailty, loneliness and social isolation.

	All (n = 461,047)	Frailty phenotype			Frailty index		
		Robust (n = 273,430)	Pre-frail (n = 172,053)	Frail (n = 15,564)	Robust (n = 293,953)	Pre-frail (n = 145,496)	Frail (n = 21,598)
Loneliness							
Not lonely	439,126 (95.2%)	264,670 (96.8%)	161,032 (93.6%)	13,424 (86.3%)	287,909 (97.9%)	133,635 (91.8%)	17,582 (81.4%)
Lonely	21,921 (4.8%)	8760 (3.2%)	11,021 (6.4%)	2140 (13.7%)	6044 (2.1%)	11,861 (8.2%)	4016 (18.6%)
Social isolation							
Not socially isolated	419,390 (91%)	254,072 (92.9%)	153,036 (88.9%)	12,282 (78.9%)	272,340 (92.6%)	129,512 (89.0%)	17,538 (81.2%)
Socially isolated	41,657 (9%)	19,358 (7.1%)	19,017 (11.1%)	3282 (21.1%)	21,613 (7.4%)	15,984 (11.0%)	4060 (18.8%)

**Figure 2:**
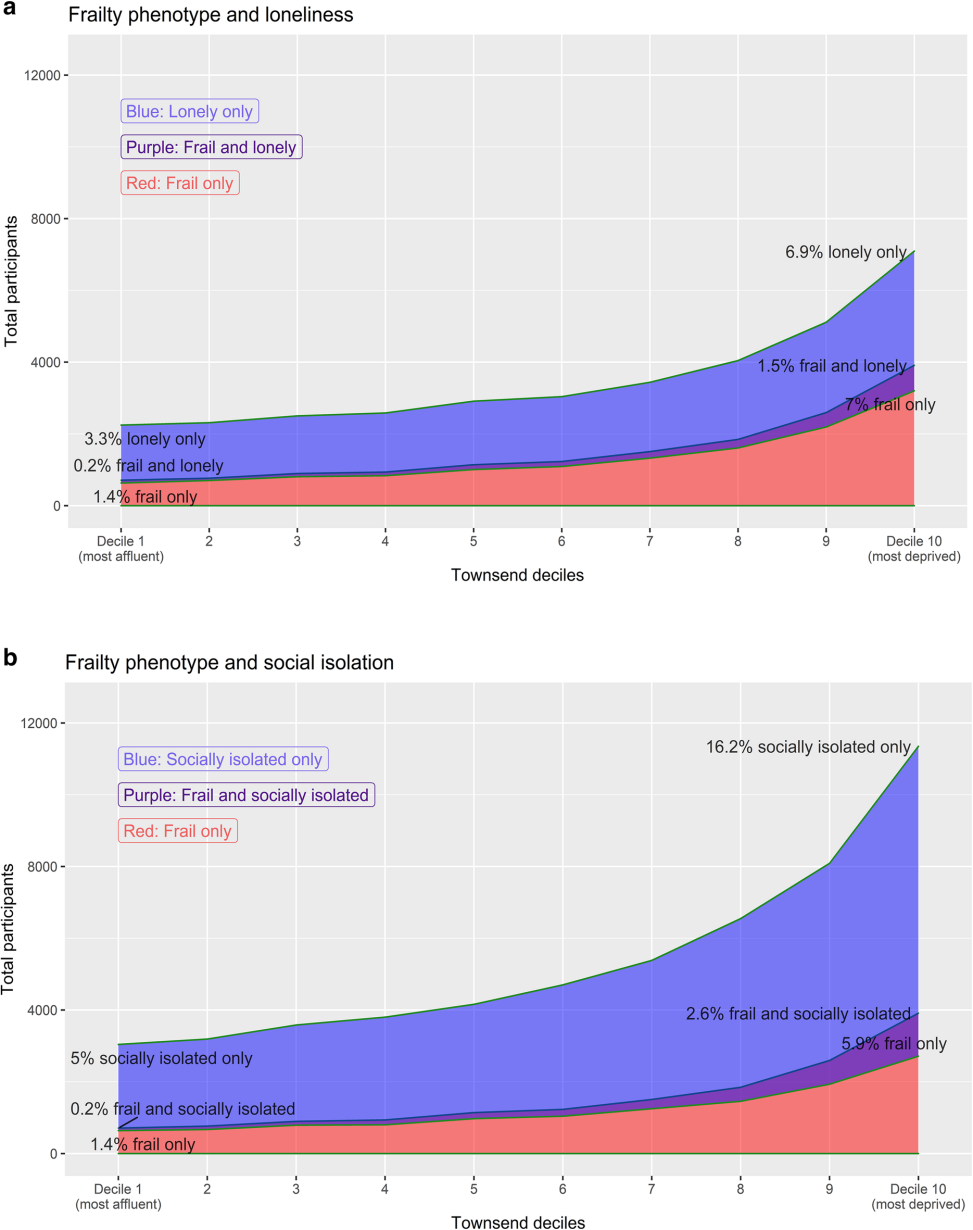
Prevalence of frailty and social isolation (1a) or loneliness (1b) by socioeconomic deprivation. Red indicates the proportion who met the criteria for frailty only, blue those who were either lonely or socially isolated (but not frail) and purple those who met the criteria for both frailty and loneliness or social isolation.

Frailty was more prevalent in women than men and among older participants, while loneliness and social isolation were more common in men and in younger participants (supplementary material). However, all three states were prevalent across the age spectrum. The combination of frailty and social isolation or loneliness was most common in the context of socioeconomic deprivation (Fig. 2).

### Association with all-cause mortality

**Figure 3:**
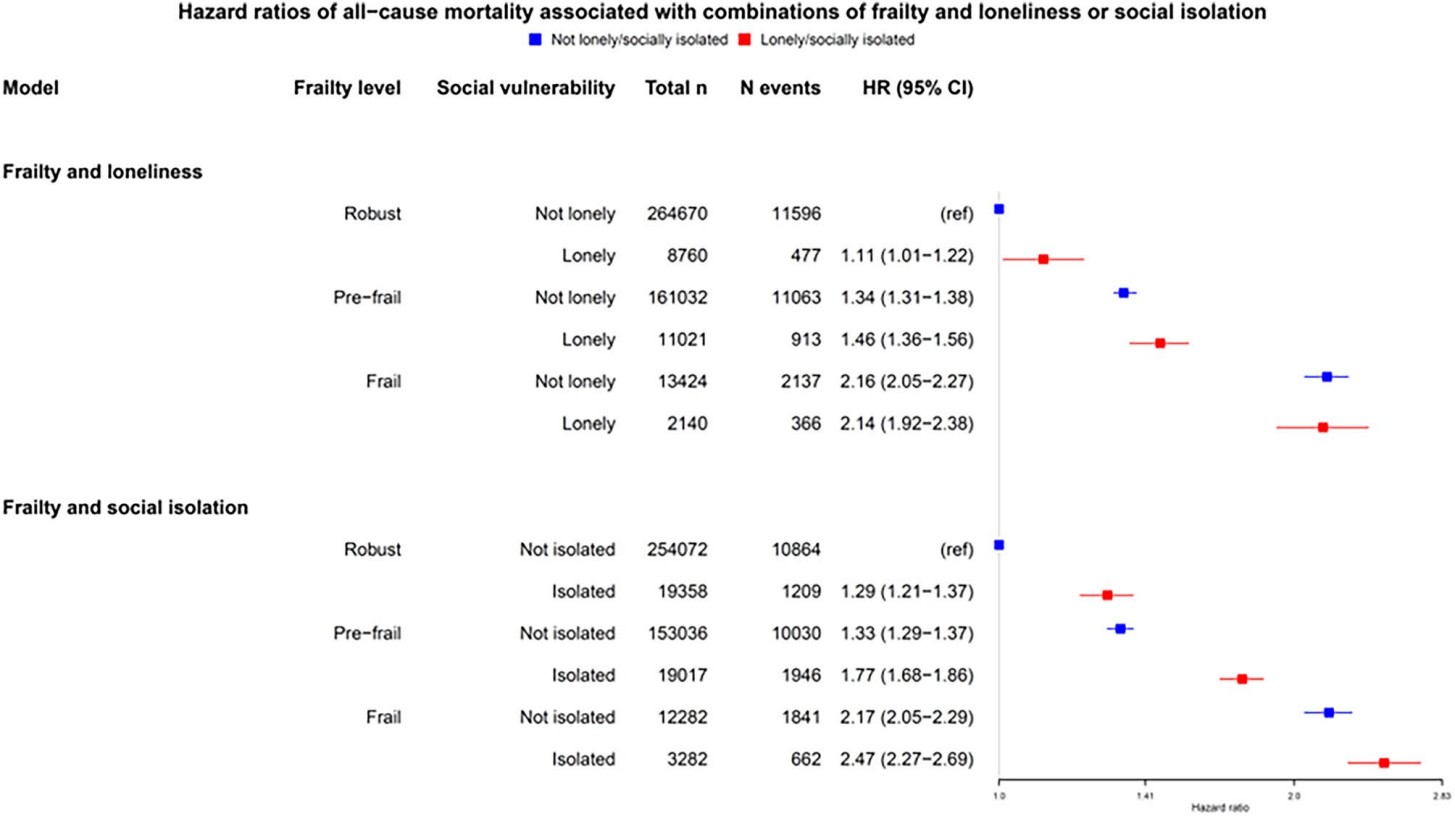
Association between combination of frailty and loneliness or social isolation and mortality. Points indicate adjusted hazard ratios. Whiskers indicate 95% confidence intervals. N = total number of participants per frailty/social vulnerability combination, n events: number of events, HR: hazard ratio, CI: confidence interval; Hazard ratios were adjusted for age, sex, ethnicity, smoking status, frequency of alcohol consumption, socioeconomic deprivation (Townsend score) and number of long-term conditions.

**Figure 4:**
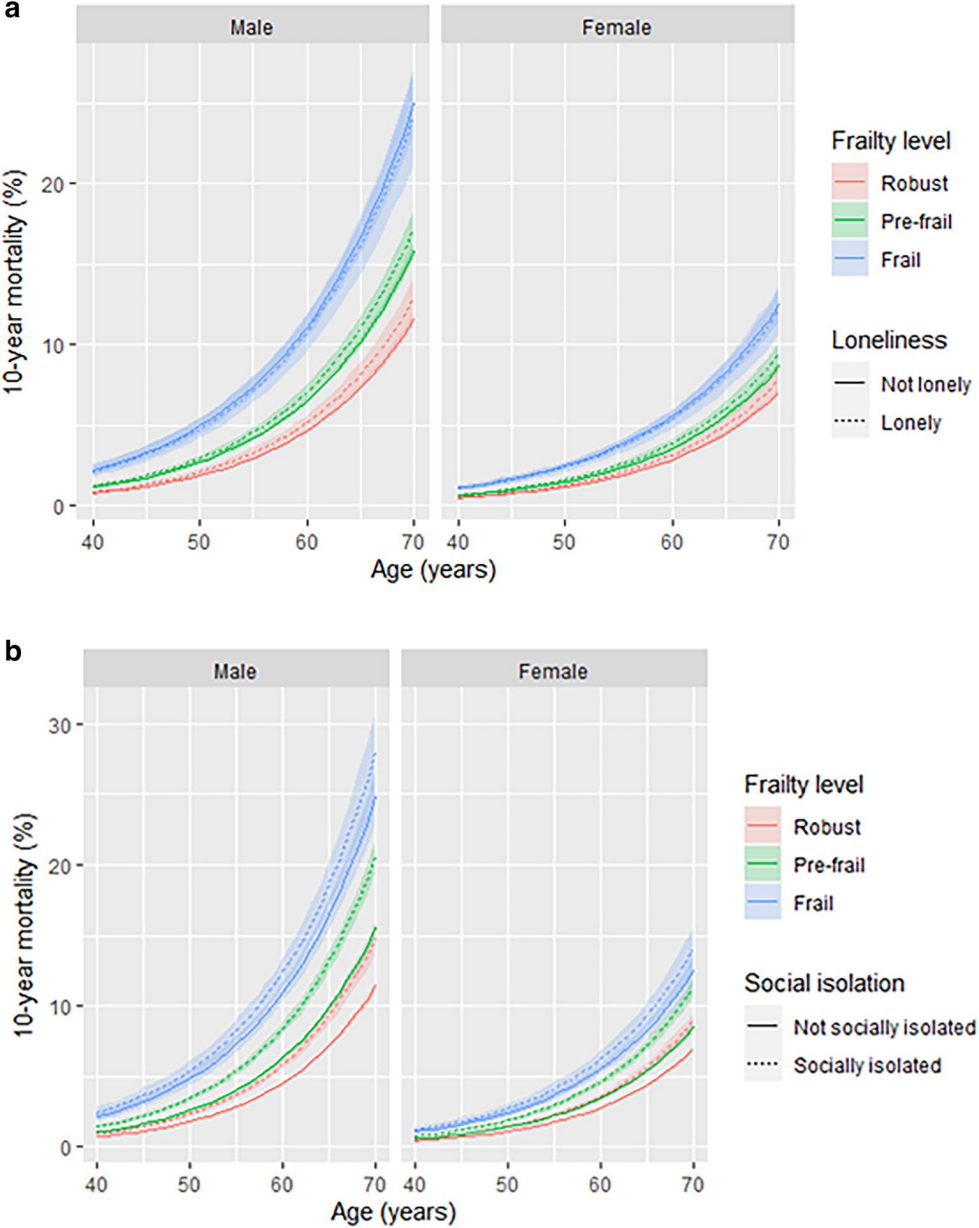
All-cause mortality—absolute risk. This figure shows the relationship between age and estimated 10-year mortality for different levels of frailty stratified by loneliness or social isolation Coloured lines indicate point estimates for predicted 10-year mortality. Colours indicate the level of frailty (red = robust, green = pre-frail, blue = frail). Shaded areas indicate 95% confidence intervals. Models are adjusted for age, sex, ethnicity, socioeconomic status, smoking, and alcohol. Predictions held socioeconomic status at the sample mean (Townsend = − 1.38) and smoking status and frequency of alcohol consumption was held at the most numerous category in the sample—‘never’ and ‘1–4 times per week’ respectively.

Hazard ratios for combinations of frailty and loneliness or social isolation and mortality in UK Biobank are shown in Fig. 3. Loneliness was associated with increased mortality risk in robust and pre-frail individuals, but not in participants with frailty. Social isolation was associated with increased mortality risk at all levels of frailty, compared with no social isolation, however the effect was smaller in people with frailty compared to pre-frail or robust (*p*-interaction < 0.01). There was no significant interaction with age, suggesting the relative association with mortality was similar across the age range included. However, on the absolute scale, the increased mortality risk associated with frailty, as well as with social isolation or loneliness, increased with age (Fig. 4) with only modest absolute increases in risk in people below 60.

Findings using the frailty index (without adjusting for multimorbidity) were similar when assessing loneliness, however social isolation was associated with increased mortality at all levels of frailty. In equivalent models using the frailty phenotype (i.e., not adjusting for multimorbidity) social isolation was associated with mortality in people with frailty.

### Association with hospitalisation

**Figure 5:**
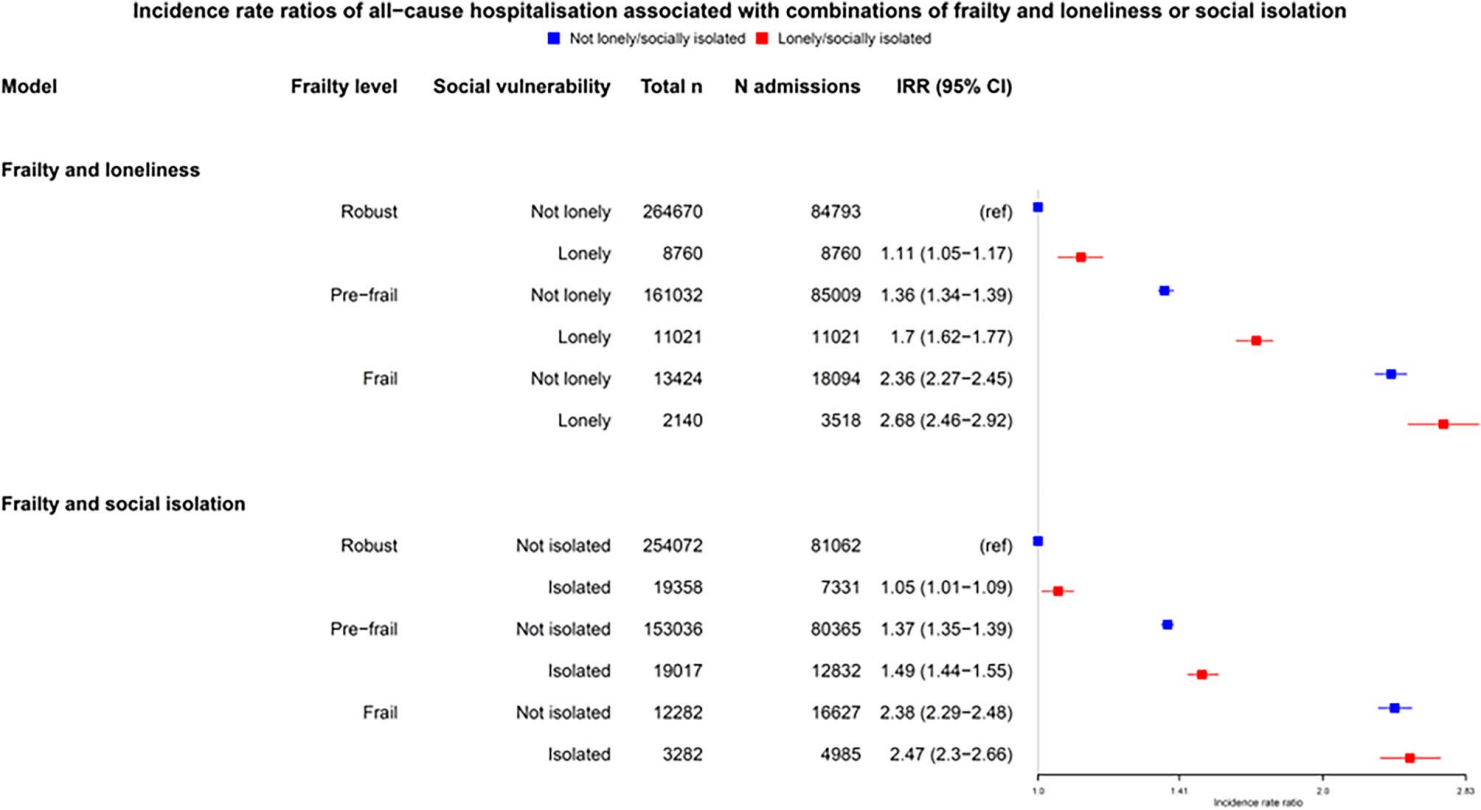
Relationship between combination of frailty and loneliness or social isolation and number of hospital admissions. Points indicate adjusted incident rate ratios. Whiskers indicate 95% CIs. N = total number of participants per frailty/social vulnerability combination, n admissions = number of admissions. CI, confidence interval; IRR, incident rate ratio. Hazard ratios were adjusted for age, sex, ethnicity, smoking status, frequency of alcohol consumption, socioeconomic deprivation (Townsend score) and multimorbidity count.

The association between combinations of frailty measure and loneliness or social isolation, and incident rate ratio for hospital admissions in are shown in Fig. 5. Loneliness was associated with greater hospitalisation risk at all levels of frailty. Social isolation was associated with increased risk in robust and pre-frail participants but was attenuated in the context of frailty. Using the frailty index, the risk associated with either loneliness or social isolation was attenuated in participants with frailty.

Similar to mortality, the absolute risk of hospitalisation was considerably higher in older people for a given level of frailty or loneliness/social isolation (supplementary appendix).

## Discussion

### Summary of findings

This analysis assessed the prevalence and impact of frailty in combination with social isolation or loneliness in UK Biobank participants aged between 37- and 73-years-old. Our findings highlight that frailty is associated with both social isolation and loneliness among relatively younger people than have been previously studied. Frailty, social isolation and loneliness are all associated with high socioeconomic deprivation: the combination of frailty with social isolation and loneliness was rare in in more affluent areas, but relatively common in more deprived communities. Finally, frailty, social isolation and loneliness are each associated with an increased risk of adverse health outcomes including mortality and hospitalisation, however the association between loneliness and social isolation and adverse outcomes was attenuated in participants with frailty.

### Comparison with other literature

The association between frailty and both loneliness and social isolation has been demonstrated across a range of countries and settings^10^. Recent systematic reviews estimate that people living with frailty were over three times more likely to experience loneliness and approximately twice as likely to be socially isolated^19,20^. The studies in these reviews focussed largely on older populations (typically > 65 years). Our findings demonstrate that these associations hold amongst younger people. Our study also expands on these previous estimates by assessing the relationship with socioeconomic deprivation, demonstrating that all three constructs are strongly associated with area-based socioeconomic position and that the combination of frailty with loneliness or social isolation is particularly more common in these settings.

Previous studies report that social vulnerability and frailty are each associated with mortality in older people^21–23^. Most of those studies assessing the combination of frailty and social vulnerability in relation to mortality have used composite measures such as the social vulnerability index (rather than loneliness or social isolation)^22,24^, with the exception of one previous study from the Netherlands^11^. This analysis builds on these by extending analysis to younger age groups. Our findings indicate that frailty, social isolation and loneliness are each associated with adverse outcomes in younger people. However, the absolute risk of mortality for a given level of frailty and loneliness or social isolation was considerably higher in older people. In people with frailty, the association between loneliness or social isolation and adverse outcomes was largely attenuated (after also adjusting for multimorbidity). This finding is similar to Amrstrong and colleagues who showed that social vulnerability (quantified using a social vulnerability index) was associated with mortality in people without frailty but that in people with a frailty index > 0.2 this association was similarly attenuated^24^. One previous study assessed the impact of combinations of frailty and social isolation or loneliness with mortality in a similar way to this study. Hoogendijk et al. studied frailty and social isolation or loneliness in people aged 65 and older in the Longitudinal Aging Study Amsterdam and, in contrast to this study, found an increased mortality risk with loneliness or social isolation in the context of frailty^11^. This difference may reflect a combination of different age ranges, differences in how loneliness and frailty were specified (the previous study used a binary frail/non-frail categorisation), different analytical choices such as covariate adjustment, or the impact of biases (such as collider bias) influencing observed associations.

Previous studies assessing the risk of hospitalisation associated with frailty and social vulnerability have not assessed loneliness or social isolation, but rather a composite measure of ‘social frailty’ which encompasses a range of these concepts^25,26^. Neither of these studies found that ‘social frailty’ was associated with hospitalisation after accounting for physical frailty.

Prevalence of combinations of frailty and loneliness/social isolation is lower than according to previous studies^11^ which may reflect the relatively younger age of UK Biobank participants, as well as the fact that UK Biobank participants are, on average, more affluent than the general population. Our finding that loneliness has a greater impact on robust/pre-frail individuals aligns with previous research which found that the association between social vulnerability and mortality was greatest among the fittest participants^24^.

Previous studies have demonstrated a complex and bidirectional relationship between frailty and both social isolation and loneliness^16,19, 27^. Our analysis was not designed to assess these trajectories, or to establish causal relationships between these constructs. Similarly, we cannot claim causal relationships between either frailty, social isolation or loneliness and mortality or hospitalisation, due to potential for residual confounding, reverse causality, and collider bias. Rather, our findings provide descriptive evidence that these states affect individuals across a wide age spectrum, co-occur in areas of high deprivation, and may identify people at greater risk of a range of adverse health outcomes who may potentially benefit from targeted intervention.

### Strengths and limitations

While this analysis used the two dominant frailty measures in the literature, as well as both subjective and objective indicators of social vulnerability, reducing some of these concepts to two/three questions may be reductive. Furthermore, the variables used to identify loneliness and social isolation are proxy measures, some of which (such as ‘living alone’ as one indicator of social isolation) may inaccurately identify participants as potentially isolated where they have other meaningful sources of social connection. Defining social isolation as the combination of at least two of these indicators, and loneliness as the presence of both explicit and implicit expressions of loneliness, is intended to minimise this limitation. Only baseline values for frailty and social vulnerability were used, however, these states may change over time and we were not able to assess these changes. Despite adjustments, there may be confounders not controlled for. Reverse causality (e.g., combined frailty and loneliness result from poor health) is also possible. There is also risk of selection bias (white, affluent participants are overrepresented in UK Biobank) which not only means that prevalence cannot be generalised and risk estimates may be conservative, but may lead to collider bias, where criteria such as UK Biobank inclusion may bias estimates of relationships between variables^28,29^. Finally, while mortality and hospitalisation could be reliably estimated and are clinically relevant, this study may not capture all relevant outcomes. For example, linkage to primary care data was not available for the full sample and is limited in its utility to identify the number and type of contact with primary care services. As a result, our analysis is limited to unscheduled hospital admissions, which can be reliably identified and measure acute hospital admissions but is an incomplete measure of overall healthcare utilisation. The impact of frailty, social isolation or loneliness, particularly among younger people, may be more fully understood by considering a wider range of outcomes such as impacts on employment, community participation, or the development of long-term health conditions.

### Implications

There is growing interest in interventions to prevent and reduce frailty or to mitigate its impact. Similarly, interventions targeting social isolation at individual, group, and policy level, with varying degrees of success, are being designed and evaluated and rolled out in a range of settings^30^. Our findings that frailty and both loneliness or social isolation frequently overlap, particularly in areas of high socioeconomic deprivation, imply that interventions focusing on one construct (e.g., frailty) must actively engage with these other issues (such as social isolation and socioeconomic deprivation) which may be barriers to recruitment, participation, retention or efficacy of interventions. For example, financial and social barriers associated with deprivation may impact an individual’s capacity to undertake nutritional or exercise-based interventions to improve frailty. Similarly, physical frailty may impede individuals' participation in group activities designed to improve social isolation. Our findings also indicate that focusing interventions purely on physical indicators (such as frailty) may neglect people with social vulnerability in less-frail groups who are also at increased risk of adverse health outcomes. The partial overlap in people identified as living with frailty (by different definitions), loneliness, or social isolation highlights a challenge in identifying these states in practice or as targets for intervention. Each of these are complex, multi-faceted states. Brief screening tools may have benefit in identifying individuals at risk (e.g. of frailty or loneliness) but may overlook or exclude some people. Furthermore, integrating identification into routine practice, often within busy and pressured healthcare systems, is challenging given the limited time and resource available to healthcare professionals and the multiple competing demands faced within healthcare. We would argue that promoting systems that value, resource and prioritise continuity of care within primary healthcare services are likely to be vital not only to recognising frailty and social vulnerability but to facilitating relational continuity and allowing for the development of therapeutic relationships responding to this need^31,32^. Finally, focussing efforts to address frailty purely on people over 65 years old risks neglecting the substantial minority of people aged under 65 who are living with frailty, most of whom live with high socioeconomic deprivation and many of whom are socially isolated. If frailty is to be prevented and ameliorated at a societal level, it is necessary to engage actively with this complexity. Such efforts must involve people living with frailty and social isolation, as well as their wider networks and communities, in designing appropriate interventions.

### Conclusion

Frailty, loneliness and social isolation are each associated with increased all-cause mortality and hospital admission in middle-aged as well as older people, however the risk associated with loneliness and social isolation is reduced in the context of frailty. Frailty and loneliness or social isolation most frequently coexist among people living with the highest levels of socioeconomic deprivation. Identification of frailty, loneliness and social isolation may provide important opportunities for intervention. However, to be successful, interventions need to consider the complex challenges which may result from combinations of physical and social vulnerability, as well as individual and structural barriers associated with deprivation.

## Methods

### Participants

From 2006 to 2010, 502,640 UK Biobank participants were recruited by postal invitation (5% response rate). Participants completed touch-screen questionnaires, interviews, and anthropometric measurements, and gave informed consent for data linkage. UK Biobank has ethical approval (NHS North West Multi-centre Research Ethics Committee (MREC): 16/NW/0274) and this analysis was performed under UK Biobank Project 14151. As the aim was to assess combinations of frailty, loneliness or social isolation, participants with missing data relating to these issues were excluded from this study. However, those with missing data on other baseline data were included in descriptive analyses. All methods were carried out in accordance with relevant guidelines and regulation (Strengthening the Reporting of Observational Studies in Epidemiology (STROBE) guidelines).

### Exposures

Exposures were frailty, which was assessed using frailty phenotype (main analysis) and frailty index (sensitivity analysis), and social isolation and loneliness. These were assessed using baseline assessment data.

#### Frailty phenotype

The phenotype model defines frailty as based on presence of the following factors: self-reported exhaustion, low grip strength, low energy expenditure, weight loss and slow gait speed. Presence of 3–5 factors constitutes frailty, 1–2 pre-frailty and zero factors classifies participants as robust^4^, as per Fried et al.’s frailty phenotype adapted for UK Biobank^2^. Detailed definitions of each criteria are described in the appendix. The higher of left- and right-hand grip strength measurements was used for grip strength and other variables were self-reported^4^.

#### Frailty index

The frailty index is a count of age-related health deficits (e.g., health conditions, symptoms, abnormal laboratory values and limitations) calculated by dividing deficits present in an individual by the total possible deficits to calculate a proportion between 0 and 1. Frailty is thus seen as the cumulative effect of individual deficits^5^. Deficits included in this measure were chosen to be those that increase in prevalence with age, are associated with poor health, and are neither too rare nor too common (i.e. < 1% prevalence in population)^33^. Deficits were selected based on the frailty index applied to UK Biobank by Williams et al.^34^ Participants were then classified as robust (frailty index < 0.12), pre-frail (0.12–0.24), or frail (> 0.24).

#### Loneliness

Loneliness was assessed using two self-reported measures, based on those in existing scales such as the revised UCLA Loneliness Scale^35^. These were “Do you often feel lonely?” (no = 0, yes = 1) and “How often are you able to confide in someone close to you?” (Almost daily to about once a month = 0, once every few months to never or almost never = 1). The two scores were summed and individuals with a score of 2 were classified as lonely. Cut-off is based on previous UK Biobank studies^18,36, 37^.

#### Social isolation

Social isolation was assessed using three self-reported measures assessing the frequency of social interaction, “Including yourself, how many people are living together in your household?” (Living alone = 1), “How often do you visit friends or family or have them visit you?” (Less than once a month = 1) and “Which of the following [activities] do you attend once a week or more often?” (None of the above = 1). Individuals with a score of 2 or 3 were classified as socially isolated. Cut-off is based on previous UK Biobank studies^18,36, 37^.

### Outcomes

#### All-cause mortality

All-cause mortality was identified from linked national mortality records (Public Health Scotland and Digital Health, England). Median follow-up duration was 11 years.

#### Hospitalisations

Number of hospital admissions classed as urgent or emergency (excluding elective admissions) were identified via record linkage to the Hospital Episode Statistics.

### Covariates

Covariates were selected as potential confounders of the relationship between frailty/social vulnerability and outcomes. These were based on baseline assessment data and were self-reported. Age and sex were used as recorded. Smoking was categorised as never, previous, or current. Self-reported alcohol intake as never/special occasions only, 1–3 times per month, 1–4 times per week, or daily/almost daily. Townsend scores were calculated from postcode areas to give an area-based measure of socioeconomic deprivation^38^. A count of long-term conditions was calculated based on 43 self-reported long-term conditions.

### Statistical analysis

All analyses are reported according to the Strengthening Reporting of Observational Studies in Epidemiology (STROBE) statement. Analysis was performed using R software (version 4.1.2).

For descriptive analyses, frailty levels (robust, pre-frail and frail) and social isolation (present/absent) or loneliness (present/absent) were cross-tabulated and the number and percentage of participants with each combination summarised. Associations between each measure and baseline sociodemographic characteristics were also summarised using counts and percentages.

Participants were categorized into groups based on their frailty and loneliness status, or frailty and social isolation status: (1) people without frailty and without loneliness, (2) people with only loneliness, (3) people with only pre-frailty, (4) people with only frailty, (5) people with pre-frailty and loneliness and (6) people with frailty and loneliness. The same was done for frailty and social isolation.

Weibull models with proportional hazards parameterisation assessed the association between the overlap of frailty and pre-frailty (for both frailty phenotype and frailty index) and loneliness or social isolation and all-cause mortality, adjusting for age, sex, ethnicity, smoking status, frequency of alcohol consumption, socioeconomic deprivation (Townsend score), and multimorbidity count. We used parametric survival models to allow us to model the baseline hazard and therefore assess mortality risk on the absolute scale, conditional on combinations of covariates. Before fitting the models, we plotted log(time) against log(-log(Kaplan Meier estimates)) for strata of each of the model variates showing linear and parallel lines for each of the covariates. This suggested that Weibull models were an appropriate fit for the data. Separate hazard ratios and 95% confident intervals were calculated for the combinations of frail and lonely or frail and socially isolated groups, with not frail, not lonely, or not frail, not socially isolated as the respective reference group depending on combination.

Statistical interactions were tested using recommendations by Knol and VanderWeele^39^ to see if impact of social isolation/loneliness varied depending on level of frailty (and vice versa) and calculated using the “epiR” R package^40^.

These models were then used to estimate the predicted 10-year risk of mortality conditional on frailty level and loneliness or social isolation as well as age and sex, in order to assess associations on the absolute scale.

Negative binomial regression models were used to model the relationship between frailty and social isolation or loneliness and number of hospital admissions (adjusting for age, sex, ethnicity, smoking status, frequency of alcohol consumption, socioeconomic deprivation and multimorbidity count). Models also included an offset term for length of follow-up to account for differential time at risk in participants who died during the follow-up period. Negative binomial models were selected over Poisson models due to overdispersion of the hospitalisation counts. We also assessed for zero-inflation using Vuong tests. Incident rate ratios and 95% confidence intervals were calculated for the combinations of frail and lonely or frail and socially isolated groups, with not frail, not lonely, or not frail, not socially isolated as the respective reference group depending on combination.

Analyses were repeated using the frailty index, however models were not adjusted for multimorbidity count as many conditions are also included in the frailty index. As such, the count of long-term conditions is intrinsic to the frailty index measure (rather than a potential confounder).

In post hoc analyses for all-cause mortality, we also assessed frailty in combination with both loneliness and social isolation.

### Supplementary Information

Supplementary Information.

## Data Availability

The UK Biobank data that support the findings of this study are available from the UK Biobank (www.ukbiobank.ac.uk), subject to approval by UK Biobank.
