# Supplementary material for: An observational analysis of frailty in combination with loneliness or social isolation and their association with socioeconomic deprivation, hospitalisation and mortality among UK Biobank participants
Source: Sci Rep. 2024 Mar 27;14:7258. doi: 10.1038/s41598-024-57366-7 (PMC10973409; doi:10.1038/s41598-024-57366-7)
Supplement: Supplementary file 1 — Supplementary Information. [file 41598_2024_57366_MOESM1_ESM.pdf]

An observational analysis of frailty in combination with loneliness or social isolation and their association with socioeconomic deprivation, hospitalisation and mortality among UK Biobank participants

Authors:

Marina Politis, Lynsay Crawford, Bhautesh D Jani , Barbara I Nicholl , Jim Lewsey , David A McAllister , Frances S Mair , Peter Hanlon

Supplementary material

Contents

Quantification of the frailty phenotype 2

Quantification of frailty index 3

Detailed baseline characteristics 5

Assessment of statistical interaction between frailty phenotype and frailty index. 8

Association of the combination of frailty (frailty phenotype), loneliness and social isolation with all-cause mortality 13

Quantification of the frailty phenotype

Taken from Hanlon P, Nicholl BI, Jani BD, Lee D, McQueenie R, Mair FS. Frailty and pre-frailty in middle-aged and older adults and its association with multimorbidity and mortality: a prospective analysis of 493 737 UK Biobank participants. The Lancet Public Health. 2018 Jul 1;3(7):e323-32.

https://doi.org/10.1016/S2468-2667(18)30091-4

Frailty phenotype variable definitions adapted for UK Biobank	
Weight loss	Self-reported: "Compared with one year ago, has your weight changed?" (response: yes, lost weight=1, other=0)
Exhaustion	Self-reported: "Over the past two weeks, how often have you felt tired or had little energy?"<br>(response: more than half the days or nearly every day=1, other=0)
Low physical activity	Self-reported: UK Biobank physical activity questionnaire. We classified the responses into: none (no physical activity in the last 4 weeks), low (light DIY activity [eg, pruning, watering the lawn] only in the past 4 weeks), medium (heavy DIY activity [eg, weeding, lawn mowing, carpentry and digging], walking for pleasure, or other exercises in the past 4 weeks), and high (strenuous sports in the past 4 weeks)<br>(response: none or light activity with a frequency of once per week or less=1, medium or heavy activity, or light activity more than once per week=0)
Slow walking pace	Self-reported: "How would you describe your usual walking pace?" (response: slow=1, other=0)
Low grip strength	Grip strength was measured using a Jamar J00105 hydraulic hand dynamometer. We took the highest value between left and right measurements for each individual and applied sex- and body-mass index adjusted cut-points for low grip strength based on the original description of the frailty phenotype by Fried et al.

Quantification of frailty index

Frailty index deficits taken from Williams DM, Jylhävä J, Pedersen NL, Hägg S. A frailty index for UK Biobank participants. The Journals of Gerontology: Series A. 2019 Mar 14;74(4):582-7.

https://doi.org/10.1093/gerona/gly094

**Deficit**	**Coding**
Glaucoma *	Categorised 0/1
Cataracts *	Categorised 0/1
Hearing difficulty	Categorised 0/1
Migraine *	Categorised 0/1
Dental problems	Categorised 0/1 for none vs. any
Self-rated health	0 – excellent; 0.25 – good; 0.5 - fair, 1 - poor
Fatigue: frequency of tiredness / lethargy in last two weeks	0, 0.25, 0.5, 1, respectively
Sleep: experience of sleeplessness/insomnia	Categorised 0, 0.5, 1, respectively
Depressed feelings: frequency in last two weeks	0 – not at all, 0.5 – several days, 0.75 -- more than half, 1 – nearly every day
Self-described nervous personality	Categorised 0/1
Severe anxiety/ panic attacks *	Categorised 0/1
Common to feel loneliness	Categorised 0/1
Sense of misery (ever/never)	Categorised 0/1
Infirmity: long-standing illness or disability	Categorised 0/1
Falls in last year	0 - no fall, 0.5 - one fall, 1 - more than one fall
Fractures/broken bones in last five years	Categorised 0/1
Diabetes *	Categorised 0/1
Myocardial infarction *	Categorised 0/1
Angina *	Categorised 0/1
Stroke *	Categorised 0/1
High blood pressure *	Categorised 0/1
Hypothyroidism *	Categorised 0/1
Deep-vein thrombosis *	Categorised 0/1
High cholesterol *	Categorised 0/1
Breathing: wheeze in last year	Categorised 0/1
Pneumonia *	Categorised 0/1
Chronic bronchitis/emphysema *	Categorised 0/1
Asthma *	Categorised 0/1
Rheumatoid arthritis *	Categorised 0/1
Osteoarthritis *	Categorised 0/1
Gout *	Categorised 0/1
Osteoporosis *	Categorised 0/1
Hayfever, allergic rhinitis or eczema *	Categorised 0/1
Psoriasis *	Categorised 0/1
Any cancer diagnosis *	Categorised 0/1
Multiple cancers diagnosed (number	Categorised 0/1

reported)	
Chest pain	Categorised 0/1
Head and/or neck pain	Categorised 0/1
Back pain	Categorised 0/1
Stomach/abdominal pain	Categorised 0/1
Hip pain	Categorised 0/1
Knee pain	Categorised 0/1
Whole-body pain	Categorised 0/1
Facial pain	Categorised 0/1
Sciatica *	Categorised 0/1
Gastric reflux *	Categorised 0/1
Hiatus hernia *	Categorised 0/1
Gall stones *	Categorised 0/1
Diverticulitis *	Categorised 0/1

Detailed baseline characteristics

			Frailty Phenotype						***Frailty Index***					
			Robust		Pre-frail		Frail		Robust		Pre-frail		Frail	
		All	Not isolated	Isolated	Not isolated	Isolated	Not isolated	Isolated	Not isolated	Isolated	Not isolated	Isolated	Not isolated	Isolated
Total		461047 (100%)	254072 (100%)	19358 (100%)	153036 (100%)	19017 (100%)	12282 (100%)	3282 (100%)	272340 (100%)	21613 (100%)	129512 (100%)	15984 (100%)	17538 (100%)	4060 (100%)
Age	Age	56.5 (8.1)	56.2 (7.8)	56.3 (8.1)	56.8 (7.8)	56.7 (7.6)	58.1 (7.5)	57.6 (8.2)	55.9 (7.9)	56 (8)	57.5 (7.8)	57 (7.5)	58.3 (8.1)	57.7 (7.8)
Sex	Female	251604 (54.6%)	133447 (52.5%)	9610 (49.6%)	88571 (57.9%)	10117 (53.2%)	8013 (65.2%)	1846 (56.2%)	144789 (53.2%)	10664 (49.3%)	74633 (57.6%)	8609 (53.9%)	10609 (60.5%)	2300 (56.7%)
	Male	209443 (45.4%)	120625 (47.5%)	9748 (50.4%)	64465 (42.1%)	8900 (46.8%)	4269 (34.8%)	1436 (43.8%)	127551 (46.8%)	10949 (50.7%)	54879 (42.4%)	7375 (46.1%)	6929 (39.5%)	1760 (43.3%)
Deprivation	Lower	153514 (33.3%)	95209 (37.5%)	4892 (25.3%)	47237 (30.9%)	3509 (18.5%)	2348 (19.1%)	319 (9.7%)	100228 (36.8%)	5254 (24.3%)	40723 (31.4%)	3004 (18.8%)	3843 (21.9%)	462 (11.4%)
	Middle	153470 (33.3%)	87985 (34.6%)	5903 (30.5%)	50644 (33.1%)	5024 (26.4%)	3301 (26.9%)	613 (18.7%)	94005 (34.5%)	6574 (30.4%)	42981 (33.2%)	4176 (26.1%)	4944 (28.2%)	790 (19.5%)
	Higher	153491 (33.3%)	70616 (27.8%)	8536 (44.1%)	54944 (35.9%)	10442 (54.9%)	6616 (53.9%)	2337 (71.2%)	77800 (28.6%)	9752 (45.1%)	45646 (35.2%)	8764 (54.8%)	8730 (49.8%)	2799 (68.9%)
	Missing	572 (0.1%)	262 (0.1%)	27 (0.1%)	211 (0.1%)	42 (0.2%)	17 (0.1%)	13 (0.4%)	307 (0.1%)	33 (0.2%)	162 (0.1%)	40 (0.3%)	21 (0.1%)	9 (0.2%)
Ethnicity	White	438852 (95.2%)	245667 (96.7%)	18412 (95.1%)	143327 (93.7%)	17505 (92%)	11011 (89.7%)	2930 (89.3%)	260362 (95.6%)	20216 (93.5%)	123187 (95.1%)	14869 (93%)	16456 (93.8%)	3762 (92.7%)
	Asian or Asian British	6998 (1.5%)	2318 (0.9%)	193 (1%)	3492 (2.3%)	358 (1.9%)	542 (4.4%)	95 (2.9%)	3809 (1.4%)	323 (1.5%)	2135 (1.6%)	253 (1.6%)	408 (2.3%)	70 (1.7%)
	Black or Black British	6620 (1.4%)	2422 (1%)	315 (1.6%)	2906 (1.9%)	499 (2.6%)	364 (3%)	114 (3.5%)	3536 (1.3%)	459 (2.1%)	1833 (1.4%)	379 (2.4%)	323 (1.8%)	90 (2.2%)
	Chinese	1156 (0.3%)	515 (0.2%)	76 (0.4%)	417 (0.3%)	102 (0.5%)	35 (0.3%)	11 (0.3%)	716 (0.3%)	133 (0.6%)	239 (0.2%)	48 (0.3%)	12 (0.1%)	8 (0.2%)
	mixed	2655 (0.6%)	1259 (0.5%)	135 (0.7%)	959 (0.6%)	187 (1%)	85 (0.7%)	30 (0.9%)	1453 (0.5%)	151 (0.7%)	746 (0.6%)	167 (1%)	104 (0.6%)	34 (0.8%)
	Other ethnic group	3501 (0.8%)	1280 (0.5%)	159 (0.8%)	1505 (1%)	268 (1.4%)	214 (1.7%)	75 (2.3%)	1800 (0.7%)	246 (1.1%)	1020 (0.8%)	185 (1.2%)	179 (1%)	71 (1.7%)
	Missing	1265 (0.3%)	611 (0.2%)	68 (0.4%)	430 (0.3%)	98 (0.5%)	31 (0.3%)	27 (0.8%)	664 (0.2%)	85 (0.4%)	352 (0.3%)	83 (0.5%)	56 (0.3%)	25 (0.6%)
Smoking	Never	251670 (54.6%)	144457 (56.9%)	9958 (51.4%)	81567 (53.3%)	8779 (46.2%)	5662 (46.1%)	1247 (38%)	159473 (58.6%)	11651 (53.9%)	64952 (50.2%)	6933 (43.4%)	7261 (41.4%)	1400 (34.5%)
	Previous	160348 (34.8%)	87839 (34.6%)	6434 (33.2%)	54317 (35.5%)	6288 (33.1%)	4426 #(36%)	1044 (31.8%)	89630 (32.9%)	6688 (30.9%)	49658 (38.3%)	5650 (35.3%)	7294 (41.6%)	1428 (35.2%)
	Current	47650 (10.3%)	21174 (8.3%)	2904 (15%)	16630 (10.9%)	3860 (20.3%)	2120 (17.3%)	962 (29.3%)	22584 (8.3%)	3197 (14.8%)	14452 (11.2%)	3326 (20.8%)	2888 (16.5%)	1203 (29.6%)
	Missing	1379 (0.3%)	602 (0.2%)	62 (0.3%)	522 (0.3%)	90 (0.5%)	74 (0.6%)	29 (0.9%)	653 (0.2%)	77 (0.4%)	450 (0.3%)	75 (0.5%)	95 (0.5%)	29 (0.7%)
Alcohol consumption	Never or special occasions only	86070 (18.7%)	33798 (13.3%)	4786 (24.7%)	33738 (22%)	6690 (35.2%)	5298 (43.1%)	1760 (53.6%)	39212 (14.4%)	5569 (25.8%)	27523 (21.3%)	5649 (35.3%)	6099 (34.8%)	2018 (49.7%)
	One to four times a week	227893 (49.4%)	135654 (53.4%)	7773 (40.2%)	73204 (47.8%)	6353 (33.4%)	4159 (33.9%)	750 (22.9%)	145337 (53.4%)	8585 (39.7%)	61075 (47.2%)	5347 (33.5%)	6605 (37.7%)	944 (23.3%)
	One to three times a month	51573 (11.2%)	25433 (10%)	2874 (14.8%)	18384 (12%)	2969 (15.6%)	1481 (12.1%)	432 (13.2%)	27947 (10.3%)	3276 (15.2%)	15165 (11.7%)	2377 (14.9%)	2186 (12.5%)	622 (15.3%)
	Daily or almost daily	95291 (20.7%)	59129 (23.3%)	3918 (20.2%)	27618 (18%)	2976 (15.6%)	1320 (10.7%)	330 (10.1%)	59763 (21.9%)	4173 (19.3%)	25672 (19.8%)	2590 (16.2%)	2632 (15%)	461 (11.4%)
	Missing	220 (0%)	58 (0%)	7 (0%)	92 (0.1%)	29 (0.2%)	24 (0.2%)	10 (0.3%)	81 (0%)	10 (0%)	77 (0.1%)	21 (0.1%)	16 (0.1%)	15 (0.4%)
Self-rated health	Excellent	77649 (16.8%)	54422 (21.4%)	3292 (17%)	18213 (11.9%)	1518 (8%)	170 (1.4%)	34 (1%)	66203 (24.3%)	4210 (19.5%)	6494 (5%)	617 (3.9%)	108 (0.6%)	17 (0.4%)
	Good	268610 (58.3%)	161587 (63.6%)	11694 (60.4%)	83695 (54.7%)	8708 (45.8%)	2482 (20.2%)	444 (13.5%)	175833 (64.6%)	13527 (62.6%)	68837 (53.2%)	6842 (42.8%)	3094 (17.6%)	477 (11.7%)
	Fair	94286 (20.5%)	35601 (14%)	3919 (20.2%)	42039 (27.5%)	6522 (34.3%)	5006 (40.8%)	1199 (36.5%)	28468 (10.5%)	3494 (16.2%)	45658 (35.3%)	6464 (40.4%)	8520 (48.6%)	1682 (41.4%)
	Poor	19010 (4.1%)	2045 (0.8%)	392 (2%)	8433 (5.5%)	2093 (11%)	4495 (36.6%)	1552 (47.3%)	1403 (0.5%)	315 (1.5%)	7930 (6.1%)	1903 (11.9%)	5640 (32.2%)	1819 (44.8%)
	Missing	1492 (0.3%)	417 (0.2%)	61 (0.3%)	656 (0.4%)	176 (0.9%)	129 (1.1%)	53 (1.6%)	433 (0.2%)	67 (0.3%)	593 (0.5%)	158 (1%)	176 (1%)	65 (1.6%)

Supplementary table 3: Baseline demographic characteristics for combinations of frailty and social isolation

			Frailty phenotype						FRAILTY INDEX					
			Robust		Pre-frail		Frail		Robust		Pre-frail		Frail	
		All	Not lonely	Lonely	Not lonely	Lonely	Not lonely	Lonely	Not lonely	Lonely	Not lonely	Lonely	Not lonely	Lonely
**Total**		461047 (100%)	264670 (100%)	8760 (100%)	161032 (100%)	11021 (100%)	13424 (100%)	2140 (100%)	287909 (100%)	6044 (100%)	133635 (100%)	11861 (100%)	17582 (100%)	4016 (100%)
**Age**		56.5 (8.1)	56.2 (8)	55.9 (8.1)	56.8 (8)	55.8 (7.6)	58.2 (7.6)	56.7 (8.1)	55.9 (8)	55.1 (7.9)	57.5 (8)	56 (7.5)	58.5 (8.1)	57.1 (8)
**Sex**	Female	251604 (54.6%)	139058 (52.5%)	3999 (45.7%)	93110 (57.8%)	5578 (50.6%)	8671 (64.6%)	1188 (55.5%)	152701 (53%)	2752 (45.5%)	77308 (57.9%)	5934 (50%)	10830 (61.6%)	2079 (51.8%)
	Male	209443 (45.4%)	125612 (47.5%)	4761 (54.3%)	67922 (42.2%)	5443 (49.4%)	4753 (35.4%)	952 (44.5%)	135208 (47%)	3292 (54.5%)	56327 (42.1%)	5927 (50%)	6752 (38.4%)	1937 (48.2%)
**Deprivation**	Lower	153514 (33.3%)	97457 (36.8%)	2644 (30.2%)	48166 (29.9%)	2580 (23.4%)	2399 (17.9%)	268 (12.5%)	103643 (36%)	1839 (30.4%)	40755 (30.5%)	2972 (25.1%)	3624 (20.6%)	681 (17%)
	Middle	153470 (33.3%)	91060 (34.4%)	2828 (32.3%)	52536 (32.6%)	3132 (28.4%)	3465 (25.8%)	449 (21%)	98649 (34.3%)	1930 (31.9%)	43603 (32.6%)	3554 (30%)	4809 (27.4%)	925 (23%)
	Higher	153491 (33.3%)	75874 (28.7%)	3278 (37.4%)	60101 (37.3%)	5285 (48%)	7539 (56.2%)	1414 (66.1%)	85288 (29.6%)	2264 (37.5%)	49101 (36.7%)	5309 (44.8%)	9125 (51.9%)	2404 (59.9%)
	Missing	572 (0.1%)	279 (0.1%)	10 (0.1%)	229 (0.1%)	24 (0.2%)	21 (0.2%)	9 (0.4%)	329 (0.1%)	11 (0.2%)	176 (0.1%)	26 (0.2%)	24 (0.1%)	6 (0.1%)
**Ethnicity**	White	438852 (95.2%)	255748 (96.6%)	8331 (95.1%)	150573 (93.5%)	10259 (93.1%)	11985 (89.3%)	1956 (91.4%)	274923 (95.5%)	5655 (93.6%)	126932 (95%)	11124 (93.8%)	16451 (93.6%)	3767 (93.8%)
	Asian or Asian British	6998 (1.5%)	2395 (0.9%)	116 (1.3%)	3610 (2.2%)	240 (2.2%)	574 (4.3%)	63 (2.9%)	4017 (1.4%)	115 (1.9%)	2159 (1.6%)	229 (1.9%)	403 (2.3%)	75 (1.9%)
	Black or Black British	6620 (1.4%)	2599 (1%)	138 (1.6%)	3180 (2%)	225 (2%)	419 (3.1%)	59 (2.8%)	3869 (1.3%)	126 (2.1%)	1984 (1.5%)	228 (1.9%)	345 (2%)	68 (1.7%)
	Chinese	1156 (0.3%)	571 (0.2%)	20 (0.2%)	492 (0.3%)	27 (0.2%)	42 (0.3%)	4 (0.2%)	824 (0.3%)	25 (0.4%)	266 (0.2%)	21 (0.2%)	15 (0.1%)	5 (0.1%)
	Mixed	2655 (0.6%)	1335 (0.5%)	59 (0.7%)	1043 (0.6%)	103 (0.9%)	100 (0.7%)	15 (0.7%)	1560 (0.5%)	44 (0.7%)	810 (0.6%)	103 (0.9%)	108 (0.6%)	30 (0.7%)
	Other ethnic group	3501 (0.8%)	1378 (0.5%)	61 (0.7%)	1659 (1%)	114 (1%)	256 (1.9%)	33 (1.5%)	1989 (0.7%)	57 (0.9%)	1104 (0.8%)	101 (0.9%)	200 (1.1%)	50 (1.2%)
	Missing	1265 (0.3%)	644 (0.2%)	35 (0.4%)	475 (0.3%)	53 (0.5%)	48 (0.4%)	10 (0.5%)	727 (0.3%)	22 (0.4%)	380 (0.3%)	55 (0.5%)	60 (0.3%)	21 (0.5%)
**Smoking**	Never	251670 (54.6%)	149975 (56.7%)	4440 (50.7%)	85156 (52.9%)	5190 (47.1%)	6072 (45.2%)	837 (39.1%)	167696 (58.2%)	3428 (56.7%)	66339 (49.6%)	5546 (46.8%)	7168 (40.8%)	1493 (37.2%)
	Previous	160348 (34.8%)	91172 (34.4%)	3101 (35.4%)	56918 (35.3%)	3687 (33.5%)	4790 (35.7%)	680 (31.8%)	94495 (32.8%)	1823 (30.2%)	51184 (38.3%)	4124 (34.8%)	7201 (41%)	1521 (37.9%)
	Current	47650 (10.3%)	22889 (8.6%)	1189 (13.6%)	18393 (11.4%)	2097 (19%)	2479 (18.5%)	603 (28.2%)	25017 (8.7%)	764 (12.6%)	15639 (11.7%)	2139 (18%)	3105 (17.7%)	986 (24.6%)
	Missing	1379 (0.3%)	634 (0.2%)	30 (0.3%)	565 (0.4%)	47 (0.4%)	83 (0.6%)	20 (0.9%)	701 (0.2%)	29 (0.5%)	473 (0.4%)	52 (0.4%)	108 (0.6%)	16 (0.4%)
**Alcohol consumption**	Never or special occasions only	86070 (18.7%)	36956 (14%)	1628 (18.6%)	37168 (23.1%)	3260 (29.6%)	6058 (45.1%)	1000 (46.7%)	43565 (15.1%)	1216 (20.1%)	30077 (22.5%)	3095 (26.1%)	6540 (37.2%)	1577 (39.3%)
	One to four times a week	227893 (49.4%)	139304 (52.6%)	4123 (47.1%)	75189 (46.7%)	4368 (39.6%)	4301 (32%)	608 (28.4%)	151104 (52.5%)	2818 (46.6%)	61457 (46%)	4965 (41.9%)	6233 (35.5%)	1316 (32.8%)
	One to three times a month	51573 (11.2%)	27206 (10.3%)	1101 (12.6%)	19823 (12.3%)	1530 (13.9%)	1637 (12.2%)	276 (12.9%)	30399 (10.6%)	824 (13.6%)	15994 (12%)	1548 (13.1%)	2273 (12.9%)	535 (13.3%)
	Daily or almost daily	95291 (20.7%)	61145 (23.1%)	1902 (21.7%)	28746 (17.9%)	1848 (16.8%)	1397 (10.4%)	253 (11.8%)	62752 (21.8%)	1184 (19.6%)	26023 (19.5%)	2239 (18.9%)	2513 (14.3%)	580 (14.4%)
	Missing	220 (0%)	59 (0%)	6 (0.1%)	106 (0.1%)	15 (0.1%)	31 (0.2%)	3 (0.1%)	89 (0%)	2 (0%)	84 (0.1%)	14 (0.1%)	23 (0.1%)	8 (0.2%)
**Self-rated health**	Excellent	77649 (16.8%)	56634 (21.4%)	1080 (12.3%)	19100 (11.9%)	631 (5.7%)	184 (1.4%)	20 (0.9%)	69247 (24.1%)	1166 (19.3%)	6571 (4.9%)	540 (4.6%)	100 (0.6%)	25 (0.6%)
	Good	268610 (58.3%)	168294 (63.6%)	4987 (56.9%)	88024 (54.7%)	4379 (39.7%)	2709 (20.2%)	217 (10.1%)	185630 (64.5%)	3730 (61.7%)	70345 (52.6%)	5334 (45%)	3052 (17.4%)	519 (12.9%)
	Fair	94286 (20.5%)	37137 (14%)	2383 (27.2%)	44248 (27.5%)	4313 (39.1%)	5439 (40.5%)	766 (35.8%)	30911 (10.7%)	1051 (17.4%)	47444 (35.5%)	4678 (39.4%)	8469 (48.2%)	1733 (43.2%)
	Poor	19010 (4.1%)	2178 (0.8%)	259 (3%)	8950 (5.6%)	1576 (14.3%)	4953 (36.9%)	1094 (51.1%)	1645 (0.6%)	73 (1.2%)	8650 (6.5%)	1183 (10%)	5786 (32.9%)	1673 (41.7%)
	Missing	1492 (0.3%)	427 (0.2%)	51 (0.6%)	710 (0.4%)	122 (1.1%)	139 (1%)	43 (2%)	476 (0.2%)	24 (0.4%)	625 (0.5%)	126 (1.1%)	175 (1%)	66 (1.6%)

Supplementary table 4: Baseline demographic characteristics for combinations of frailty and loneliness

Assessment of statistical interaction between frailty phenotype and frailty index.

Statistical interactions were tested using recommendations by Knol and VanderWeele to see if impact of social isolation/loneliness varied depending on level of frailty (and vice versa) and calculated using the "epiR" R package. Presence of interactions on additive scale meant that combined effects of two exposures is larger or smaller than the sum of their individual effects whereas interactions on the multiplicative scale means that combined effect is larger/smaller than the product of individual effects. Additive interactions were measured via synergy index (SI) and multiplicative interactions via ratio of hazard ratios (HRs) or incident rate ratios (IRRs). 95% Confidence Intervals (95% CI) were also calculated.

These analyses were not adjusted for multimorbidity count, as many long term conditions were common to both the frailty index and the morbidity count.

Knol MJ, VanderWeele TJ. Recommendations for presenting analyses of effect modification and interaction. Int J Epidemiol. 2012;41(2):514-20.

Stevenson M, Nunes, T., Heuer, C., Marshall, J., Sanchez, J., Thornton, R. et al 2018. epiR: Tools for the Analysis of Epidemiological Data, R Package Version 0; pp. 9–97. epiR: Tools for the Analysis of Epidemiological Data, R Package Version 0; pp. 9–97. 2018.

Interaction between loneliness (not lonely, lonely) and frailty phenotype (robust, pre-frail, frail) on all-cause mortality

		Frailty phenotype						ORs (95%CI) for pre-frail within strata of loneliness	ORs (95%CI) for frail within strata of loneliness
		Robust		Pre-frail		Frail			
		N	HR (95%CI)	N	HR (95%CI)	N	HR (95%CI)		
Loneliness	Not lonely	264670	1.0	161032	1.46 (1.42 – 1.50); p= < 0.001	13424	2.9 (2.76 – 3.04); p= <0.001	1.46 (1.42 – 1.50); p= < 0.001	2.9 (2.76 – 3.04); p= <0.001
	Lonely	8760	1.14 (1.04 – 1.25); p=0.005	11021	1.67 (1.56 – 1.79); p= < 0.001	2140	2.94 (2.64 – 3.27); p= < 0.001	1.46 (1.31-1.64); p=<0.001	2.57 (2.24-2.96); p=<0.001
HRs (95%CI) for loneliness within strata of frailty phenotype			1.14 (1.04 – 1.25); p=0.005		1.14 (1.07-1.22); p=<0.001		1.02 (0.91-1.14); p=0.787		
Measure of interaction on additive scale: Synergy index (95%CI)					1.11 (0.87-1.42)		0.95 (0.80-1.14)		
	RERI (95%CI)				0.07 (-0.09 – 0.22)		-0.10 (-0.44-0.25)		
Measure of interaction on multiplicative scale: Ratio of HRs (95%CI)					1.00 (0,89-1.12)		0.89 (0.77-1.03)		

ORs are adjusted for ethnicity, age, sex, deprivation (Townsend score), frequency of alcohol consumption, smoking status

Interaction between social isolation (not socially isolated, socially isolated) and frailty phenotype (robust, pre-frail, frail) on all-cause mortality

		Frailty phenotype						ORs (95%CI) for pre-frail within strata of social isolation	ORs (95%CI) for frail within strata of social isolation
		Robust		Pre-frail		Frail			
		N	OR (95%CI)	N	OR (95%CI)	N	OR (95%CI)		
Social isolation	Not socially isolated	254072	1.0	153036	1.45 (1.41-1.49); p=<0.001	12282	2.89 (2.75-3.05); p=<0.001	1.45 (1.41-1.49); p=<0.001	2.89 (2.75-3.05); p=<0.001
	Socially isolated	19358	1.29 (1.22-1.37); p=<0.001	19017	1.96 (1.87-2.06); p=<0.001	3282	3.38 (3.11-3.67); p=<0.001	1.52 (1.41-1.63); p=<0.001	2.61 (2.37-2.88); p=
ORs (95%CI) for social isolation within strata of frailty index			1.29 (1.22-1.37); p=<0.001		1.36 (1.29-1.42); p=<0.001		1.17 (1.07-1.28); p=<0.001		
Measure of interaction on additive scale: Synergy index (95%CI)					1.30 (1.13 – 1.50)		1.09 (0.96-1.24)		
	RERI (95%CI)				0.22 (0.10 – 0.34)		0.19 (-0.11-0.50)		
Measure of interaction on multiplicative scale: Ratio of ORs (95%CI)					1.05 (0.97 – 1.13)		0.90 (0.81-1.01)		

ORs are adjusted for ethnicity, age, sex, deprivation (Townsend score), frequency of alcohol consumption, smoking status

Interaction between loneliness (not lonely, lonely) and frailty index (robust, pre-frail, frail) on all-cause mortality

		Frailty Index						ORs (95%CI) for pre-frail within strata of loneliness	ORs (95%CI) for frail within strata of loneliness
		Robust		Pre-frail		Frail			
		N	OR (95%CI)	N	OR (95%CI)	N	OR (95%CI)		
Loneliness	Not lonely	287909	1	133635	1.47 (1.43-1.51); p=<0.001	17582	2.25 (2.15-2.36); p=<0.001	1.47 (1.43-1.51); p=<0.001	2.25 (2.15-2.36); p=<0.001
	Lonely	6044	1.15 (1.03-1.29); p=0.017	11861	1.46 (1.36-1.56); p=<0.001	4016	2.31 (2.12-2.52); p=<0.001	1.27 (1.11-1.45); p=<0.001	2.01 (1.75-2.31); p=<0.001
ORs (95%CI) for loneliness within strata of frailty index			1.15 (1.03-1.29); p=0.017		0.99 (0.93-1.07); p=0.856		1.02 (0.93-1.12); p=0.618		
Measure of interaction on additive scale: Synergy index (95%CI)					0.74 (0.55 – 1.01)		0.93 (0.77 – 1.12)		
RERI (95%CI)					-0.16 (-0.32 – 0,01)		-0.09 (-0.34 – 0.16)		
Measure of interaction on multiplicative scale: Ratio of ORs (95%CI)					0.86 (0.76 – 0.99)		0.89 (0.77 – 1.03)		

ORs are adjusted for ethnicity, age, sex, deprivation (Townsend score), frequency of alcohol consumption, smoking status

Interaction between socially isolation (not socially isolated, socially isolated) and frailty index (robust, pre-frail, frail) on all-cause mortality

		Frailty Index						ORs (95%CI) for pre-frail within strata of social isolation	ORs (95%CI) for frail within strata of social isolation
		Robust		Pre-frail		Frail			
		N	OR (95%CI)	N	OR (95%CI)	N	OR (95%CI)		
Social Isolation	Not socially isolated	272340	1.0	129512	1.45 (1.41-1.49); p=<0.001	17538	2.21 (2.11-2.32); p=<0.001	1.45 (1.41-1.49); p=<0.001	2.21 (2.11-2.32); p=<0.001
	Socially isolated	21613	1.30 (1.23-1.38); p=<0.001	15984	1.95 (1.85-2.06); p=<0.001	4060	2.90 (2.68-3.13); p=<0.001	1.50 (1.40-1.61); p=<0.001	2.23 (2.03-2.44); p=<0.001
ORs (95%CI) for social isolation within strata of frailty index			1.30 (1.23-1.38); p=<0.001		1.35 (1.28-1.42); p=<0.001		1.31 (1.20-1.42); p=<0.001		
Measure of interaction on additive scale: Synergy index (95%CI)					1.27 (1.10 – 1.47)		1.25 (1.09 – 1.44)		
RERI (95%CI)					0.21 (0.08 – 0.33)		0.38 (0.14 – 0.63)		
Measure of interaction on multiplicative scale: Ratio of ORs (95%CI)					1.04 (0.96 – 1.12)		1.01 (0.91 – 1.11)		

ORs are adjusted for ethnicity, age, sex, deprivation (Townsend score), frequency of alcohol consumption, smoking status

Interaction between loneliness (not lonely, lonely) and frailty phenotype (robust, pre-frail, frail) on number of hospitalisations

		Frailty phenotype<br>Robust<br>IRR (95%CI)	Pre-frail<br>IRR (95%CI)	Frail<br>IRR (95%CI)	IRRs (95%CI) for pre-frail within strata of loneliness	IRRs (95%CI) for frail within strata of loneliness
Loneliness	Not lonely	1.0	1.57 (1.54-1.59); p=<0.001	3.69 (3.55-3.83); p=<0.001	1.57 (1.54-1.59); p=<0.001	3.69 (3.55-3.83); p=<0.001
	Lonely	1.17 (1.11-1.24); p=<0.001	2.08 (1.99-2.17); p=<0.001	O4.42 (4.04-4.83); p=<0.001	1.77 (1.65-1.89); p=<0.001	3.76 (3.39-4.16); p=<0.001
IRRs (95%CI) for loneliness within strata of frailty phenotype		1.17 (1.11-1.24); p=<0.001	1.33 (1.27-1.38); p=<0.001	1.20 (1.09-1.32); p=<0.001		
Measure of interaction on additive scale: Synergy index (95%CI)			1.45 (1.24-1.69)	1.19 (1.02 -1.40)		
RERI (95%CI)			0.33 (0.20 – 0.47)	0.55 (0.02 – 1.08)		
Measure of interaction on multiplicative scale: Ratio of IRRs (95%CI)			1.13 (1.03 – 1.23)	1.02 (0.88 – 1.18)		

IRRs are adjusted for ethnicity, age, sex, deprivation (Townsend score), frequency of alcohol consumption, smoking status

Interaction between social isolation (not socially isolated, socially isolated) and frailty phenotype (robust, pre-frail, frail) on number of hospitalisations

		Frailty phenotype<br>Robust<br>IRR (95%CI)	Pre-frail<br>IRR (95%CI)	Frail<br>IRR (95%CI)	IRRs (95%CI) for pre-frail within strata of social isolation	IRRs (95%CI) for frail within strata of social isolation
Social isolation	Not socially isolated	1.0	1.58 (1.55-1.60); p=<0.001	3.75 (3.60-3.90); p=<0.001	1.58 (1.55-1.60); p=<0.001	3.75 (3.60-3.90); p=<0.001
	Socially isolated	1.06 (1.02-1.10); p=0.003	1.78 (1.71-1.84); p=<0.001	3.93 (3.66-4.23); p=<0.001	1.68 (1.60-1.76); p=<0.001	3.72 (3.43-4.03); p=<0.001
IRRs (95%CI) for social isolation within strata of frailty index		1.06 (1.02-1.10); p=0.003	1.13 (1.09-1.17); p=<0.001	1.05 (0.97-1.14); p=0.242		
Measure of interaction on additive scale: Synergy index (95%CI)			1.22 (1.07 – 1.39)	1.05 (0.91 – 1.20)		
RERI			0.14 (0.05 – 0.23)	0.13 (-0.28 -0.53)		
Measure of interaction on multiplicative scale: Ratio of IRRs (95%CI)			1.06 (1.00 – 1.14)	0.99 (0.88-1.12)		

IRRs are adjusted for ethnicity, age, sex, deprivation (Townsend score), frequency of alcohol consumption, smoking status

Interaction between loneliness (not lonely, lonely) and frailty index (robust, pre-frail, frail) on number of hospitalisations

		Frailty Index			IRRs (95%CI) for pre-frail within strata of loneliness	IRRs (95%CI) for frail within strata of loneliness
		Robust	Pre-frail	Frail		
		IRR (95%CI)	IRR (95%CI)	IRR (95%CI)		
Loneliness	Not lonely	1.0	1.87 (1.84-1.90); p=<0.001	3.95 (3.83-4.08); p=<0.001	1.87 (1.84-1.90); p=<0.001	3.95 (3.83-4.08); p=<0.001
	Lonely	1.09 (1.02-1.16); p=0.012	1.95 (1.87-2.03); p=<0.001	3.98 (3.73-4.25); p=<0.001	1.79 (1.66-1.93); p=<0.001	3.66 (3.34-4.01); p=<0.001
IRRs (95%CI) for loneliness within strata of frailty index		1.09 (1.02-1.16); p=0.012	1.04 (1.00-1.08); p=0.083	1.01 (0.94-1.08); p=0.835		
Measure of interaction on additive scale: Synergy index (95%CI)			0.98 (0.85-1.14)	0.98 (0.87 – 1.11)		
	RERI(95%CI)		-0.02 (-0.16-0.13)	-0.06 (-0.43 – 0.32)		
Measure of interaction on multiplicative scale: Ratio of IRRs (95%CI)			0.95 (0.86 -1.06)	0.93 (0.82 -1.05)		

IRRs are adjusted for ethnicity, age, sex, deprivation (Townsend score), frequency of alcohol consumption, smoking status

Interaction between socially isolation (not socially isolated, socially isolated) and frailty index (robust, pre-frail, frail) on number of hospitalisations

		Frailty Index			IRRs (95%CI) for pre-frail within strata of social isolation	IRRs (95%CI) for frail within strata of social isolation
		Robust	Pre-frail	Frail		
		IRR (95%CI)	IRR (95%CI)	IRR (95%CI)		
Social Isolation	Not socially isolated	1.0	1.87 (1.84-1.90); p=<0.001	3.93 (3.81-4.06); p=<0.001	1.87 (1.84-1.90); p=<0.001	3.93 (3.81-4.06); p=<0.001
	Socially isolated	1.09 (1.05-1.12); p=<0.001	2.02 (1.95-2.09); p=<0.001	4.23 (3.97-4.52); p=<0.001	1.86 (1.77-1.95); p=<0.001	3.90 (3.63-4.19); p=<0.001
IRRs (95%CI) for social isolation within strata of frailty index		1.09 (1.05-1.12); p=<0.001	1.08 (1.04-1.12); p=<0.001	1.08 (1.00-1.15); p=0.037		
Measure of interaction on additive scale: Synergy index (95%CI)			1.06 (0.95 – 1.18)	1.07 (0.95 -1.21)		
	RERI (95%CI)		0.06 (-0.05 – 0.17)	0/23 (-0.17 -0.14)		
Measure of interaction on multiplicative scale: Ratio of IRRs (95%CI)			0.99 (0.93 – 1.06)	0.99 (0.90 – 1.10)		

IRRs are adjusted for ethnicity, age, sex, deprivation (Townsend score), frequency of alcohol consumption, smoking status

Association of the combination of frailty (frailty phenotype), loneliness and social isolation with all-cause mortality

Frailty/loneliness/social isolation combination	HR	LCI	UCI
Robust, no loneliness, no social isolation	Ref	-	-
Robust, lonely, no social isolation	1.08	0.96	1.2
Robust, no loneliness, socially isolated	1.27	1.2	1.36
Robust, lonely and socially isolated	1.51	1.28	1.8
Pre-frail, no loneliness, no social isolation	1.44	1.4	1.48
Pre-frail, lonely, no social isolation	1.64	1.51	1.78
Pre-frail, no loneliness, socially isolated	1.97	1.87	2.08
Pre-frail, lonely and socially isolated	1.95	1.74	2.18
Frail, no loneliness, no social isolation	2.9	2.74	3.06
Frail, lonely, no social isolation	2.95	2.57	3.39
Frail, no loneliness, socially isolated	3.43	3.13	3.77
Frail, lonely and socially isolated	3.27	2.78	3.84
HR: Hazard ratio<br>LCI: Lower 95% confidence interval<br>UCI: Upper 95% confidence interval			
